# Supplementary material for: MetaRibo-Seq measures translation in microbiomes
Source: Nat Commun. 2020 Jun 29;11:3268. doi: 10.1038/s41467-020-17081-z (PMC7324362; doi:10.1038/s41467-020-17081-z)
Supplement: Supplementary file 10 — Supplementary Data 7 [file 41467_2020_17081_MOESM10_ESM.zip › File2/Confidence_High_Taxonomy/158861_out.krona.html]

Javascript must be enabled to view this page.

members
magnitude
magnitudeUnassigned
count
unassigned
taxon
rank

158861\_out

8

8
2
superkingdom

phylum
8
1239

186801
8
class


SRS064757\_contig\_number\_contig-100\_1344.1345
order
1
8
186802

7
1
186803
family

SRS015794\_contig\_number\_8480

4
189330
genus

species

SRS148091\_contig\_number\_11317
1262873
1

species

SRS015065\_contig\_number\_28895SRS017521\_contig\_number\_37788SRS019068\_contig\_number\_48780
3
88431

1506577
1
genus

29361
1
species

SRS1055043\_contig\_number\_contig-100\_634.158745

572511
1
genus

1
33035

SRS148159\_contig\_number\_contig-100\_2169.313258
species
